# Presence of serum amyloid A3 in mouse plasma is dependent on the nature and extent of the inflammatory stimulus

**DOI:** 10.1038/s41598-020-66898-7

**Published:** 2020-06-25

**Authors:** Alan Chait, Laura J. den Hartigh, Shari Wang, Leela Goodspeed, Ilona Babenko, William A. Altemeier, Tomas Vaisar

**Affiliations:** 1 Divisions of Metabolism, Endocrinology and Nutrition, University of Washington, Seattle, WA 98109 USA; 2 Pulmonary, Critical Care and Sleep Medicine, University of Washington, Seattle, WA 98109 USA

**Keywords:** Cell biology, Molecular biology, Endocrinology

## Abstract

Serum amyloid A3 (Saa3) derives mainly from extrahepatic tissue and is not detected in plasma from moderately inflamed obese mice. In contrast, it is present in plasma from mice acutely inflamed by injection of high dose of lipopolysaccharide (LPS). To reconcile these differences, we evaluated whether different acute inflammatory stimuli could affect the presence of Saa3 in plasma. Saa3 appeared dose dependently in plasma after LPS injection. In contrast, only very low levels were detected after sterile inflammation with silver nitrate despite levels of Saa1 and Saa2 being comparable to high dose LPS. Saa3 was not detected in plasma following casein administration. Although most Saa3 was found in HDL, a small amount was not lipoprotein associated. Gene expression and proteomic analysis of liver and adipose tissue suggested that a major source of Saa3 in plasma after injection of LPS was adipose tissue rather than liver. We conclude that Saa3 only appears in plasma after induction of acute inflammation by some but not all inflammatory stimuli. These findings are consistent with the observation that Saa3 is not detectable in plasma in more moderate chronic inflammatory states such as obesity.

## Introduction

The serum amyloid A (Saa) family of proteins are comprised of 4 major forms, Saa1, Saa2 and Saa3, which are acute phase proteins, and Saa4, which is constitutively expressed. Saa1 and Saa2, increase dramatically and rapidly in plasma following acute inflammatory stimuli^1^, but also are increased more modestly and chronically in chronic inflammatory conditions such as obesity^2–5^. Saa1 and Saa2 are predominantly produced by the liver, whereas Saa3 is commonly regarded as an extrahepatic form of Saa secreted by cells such as macrophages and adipocytes^1^ in species such as mice, although it also can be made at low levels by hepatocytes^6,7^. However, *SAA3* is a pseudogene in humans and some primates because of a frameshift mutation that generates a downstream stop signal, such that no *SAA3* mRNA or SAA3 protein has been identified in humans^1^. Unlike mice, the isotype of SAA produced by extrahepatic cells such as inflamed macrophages and adipocytes is SAA1 and SAA2, which is the same isotype as produced by the liver under inflammatory conditions^1^. There is evidence that extrahepatic SAA exerts functions that may be distinct from hepatic SAA^8,9^. For example, Saa1, Saa2 and Saa3 have distinct systemic and local functions in promoting Th17-mediated inflammatory diseases^9^. Therefore, we have taken advantage of the different isotypes of Saa produced by hepatic (Saa1 and Saa2) and extrahepatic tissues (Saa3) in mice to specifically study the role of extrahepatic Saa. The isotype difference between mice and humans provides us with a unique and the fortuitous ability to specifically study extrahepatic Saa.

The SAA family of proteins are apolipoproteins that are transported in plasma predominantly bound to high density lipoproteins (HDL)^10,11^. However, in certain mouse models^12,13^ and some obese humans^14^, some SAA can also be transported in lower density lipoproteins. Because Saa3 only has about 65% homology with Saa1 and Saa2, it can be readily distinguished from Saa1 and Saa2. Therefore, studying the expression and secretion of Saa3 in inflamed mice can provide important insights into the regulation and function of extrahepatically-derived Saa. Using highly specific and sensitive mass spectrometric techniques, we previously showed that Saa3 did not contribute to circulating Saa levels in obese mice^5^. However, Tannock *et al*. recently demonstrated the presence of small amounts of Saa3 in HDL from mice injected with high doses of lipopolysaccharide (LPS)^6^. To examine whether the nature and degree of the inflammatory stimulus determines whether Saa3 is detectable in plasma, we employed several modalities of acute inflammation in mice previously shown to elevate circulating levels of Saa.

Using LC-MS/MS, a highly specific and sensitive method of detection, we found that high dose LPS resulted in the appearance of low levels of Saa3 in plasma, with lower doses of LPS and sterile inflammation induced by silver nitrate leading to very low levels of Saa3, while resulting in high levels of Saa1 and Saa2. Saa3 was undetectable in plasma after the other inflammatory stimuli tested. Although most Saa3 found in plasma was in HDL, after high dose LPS a small amount of Saa3 was detected in FPLC fractions corresponding to molecular sizes smaller than HDL. Based on gene expression and proteomic analysis in adipose tissue and liver, the source of the plasma Saa3 after high dose LPS appears to be largely from adipose tissue.

## Materials and Methods

### Mouse study design

Male C57Bl/6 mice or *Saa3*^*−/−*^ mice on the C57Bl/6 background (described in^7^) between 2–4 months of age were injected with either LPS (intraperitoneal, 0.25, 2.5, or 25 µg, E Coli 0111:B4, List Biological Laboratory), silver nitrate (AgNO_3_, subcutaneous, 0.5 mL of a 1% solution, Sigma), or casein (subcutaneous, 0.5 mL of a 5% solution) (n = 3 mice/group). *Saa3* KO mice were generated by injecting *Saa3*-null embryonic stems cells into albino C57BL/6 embryos as previously described^7^. Injection-free mice were used as controls to eliminate any inflammation that might be elicited by a control injection. Injection doses were chosen based on a previous study^15^. The selected times were based on a previous report showing that Saa1, Saa2, and Saa3 expression levels are maximal between 16–29 h following LPS or casein injection^15,16^. For all cohorts of mice, blood was collected and PBS-perfused epididymal white adipose tissue (EWAT) and liver were collected and snap frozen in liquid nitrogen. All experimental procedures were approved by the University of Washington Institutional Animal Care and Use Committee and followed the NIH guidelines for the care and use of laboratory animals (NIH publication no. 8023, revised 1978).

### Plasma analyses

A sample of 100 µL of plasma was subjected to fast protein liquid chromatography (FPLC) on a Superose 6 10/300GL size exclusion column (GE Healthcare Life Sciences) in phosphate-buffered saline at a flow rate of 0.4 mL/min, and fractions were collected in 0.5 mL increments. Cholesterol was quantified from collected fractions using a colorimetric assay as previously described^12^. Total Saa1/2 was quantified in plasma by ELISA (R&D Systems)^7^.

### Gene expression analysis

EWAT and liver samples were extracted with commercially available RNA extraction kit (Qiagen RNeasy Mini Kit) to obtain total RNA. RNA was spectroscopically quantified and 2 µg of RNA were reverse-transcribed. The resulting cDNA was quantified using real-time quantitative PCR by standard protocols on an ABI 7900HT instrument. Primer and probe sets for measured genes were purchased from Thermo Fisher Scientific (TaqMan system). We used *Gapdh* as a reference gene. The various treatments did not affect levels of *Gapdh*. Moreover they were similar between liver and adipose tissue. Relative amounts of the measured genes were calculated using the ΔΔCt formula. For analysis they were expressed as a fold change referenced to control EWAT. Table 1 summarized accession numbers for Taqman primers used.

**Table 1:** Accession numbers for Taqman primers.

Gene Name	Thermo Fisher Scientific accession number
*Gapdh*	Mm99999915_g1
*Saa1*	Mm00656927_g1
*Saa2*	Mm04208126_mH
*Saa3*	Mm00441203_m1

### Mass spectrometry

#### Plasma digestion

Digestion of plasma samples was performed as follows – 3 µL of plasma was diluted 20x in 100 mM NH_4_HCO_3_. The diluted plasma (10 µL) was further diluted to 50 µL with 0.625% sodium deoxycholate (SDC) in 100 mM NH_4_HCO_3_ and spiked with 0.05 µg of ^15^N-APOA1 and a mixture of stable isotope labeled peptides (SIL) for the following proteins – Apoa1, Saa1, Saa2, Saa3, and Apoc3 (list of peptides are shown in Table 2). The samples were reduced with dithiothreitol (DTT) (30 min at 60 °C) and alkylated with iodoacetamide (IAA) (45 min at room temperature in the dark). To remove unreacted IAA, more DTT was added and the samples digested with 3 µg of trypsin (Worthington Biochemical Corp, Lakewood, NJ) (~1:10 w/w to plasma protein). SDC was removed after precipitation with 5 µL of 20% trifluoroacetic acid and centrifugation. Sixty µL was transferred to a PCR plate and frozen at −20 °C until analysis.

**Table 2:** Peptides used in quantification of the measured proteins.

Protein	Peptide	Data Processing	Sequence position	Precursor ion	Precursor Charge
Saa3	R.ADQFANEWGR.S	SIL	[95,104]	597.27	2+
Saa1	R.EAFQEFFGR.G	SIL	[80,88]	565.77	2+
Saa2	R.ESFQEFFGR.G	SIL	[80,88]	573.76	2+
Saa4	R.EAVQGTWDLWR.A	Prot. Average	[26,36]	680.84	2+
Saa4	R.DNLEANYQNADQYFYAR.G	Prot. Average	[40,56]	698.98	3+
Saa4	K.YFQGLLNR.Y	Prot. Average	[81,88]	505.77	2+
Apoa1	K.VQPYLDEFQK.K	SIL	[119,128]	633.82	2+
Apoc3	K.TVQDALSSVQESDIAVVAR.G	SIL	[41,59]	663.35	3+
Apoe	K.ELEEQLGPVAEETR.A	Prot. Average	[86,99]	800.40	2+
Apoe	R.LGPLVEQGR.Q	Prot. Average	[190,198]	484.78	2+
Apoe	R.TANLGAGAAQPLR.D	Prot. Average	[201,213]	620.34	2+
Alb	K.LVQEVTDFAK.T	Prot. Average	[65,74]	575.31	2+
Alb	K.LCAIPNLR.E	Prot. Average	[97,104]	478.77	2+
Alb	K.APQVSTPTLVEAAR.N	Prot. Average	[438,451]	720.40	2+
Apoa2	K.TSEIQSQAK.A	Prot. Average	[53,61]	496.26	2+
Apoa2	K.THEQLTPLVR.S	Prot. Average	[67,76]	597.34	2+

SIL - Stable isotope labeled peptide used to calculate response.

Prot. Average - three peptides averaged to determine protein response.

#### FPLC fraction digestion

The same volume of FPLC fractions (corresponding to maximum of 10 μg of protein determined using the Bradford assay) was digested following the same protocol as for plasma, including spiking with ^15^N-APOA1 and SIL peptides.

#### LC-MS/MS analysis

The abundance of selected proteins was quantified by mass spectrometry using data independent analysis (DIA). After desalting on a C18 trapping column (Reprosil-Pur 120 C18-AQ, 5 µm, 0.1 × 40 mm, Dr. Maisch HPLC GmbH, Germany) (flow rate 4 μL/min), the digested peptides were separated on an analytical column (Reprosil-Pur 120 C18-AQ, 5 µm, 250 × 0.075 mm, Dr. Maisch HPLC GmbH). Following multi-step linear gradient was used: 1–5%B in 2 min, 5–25% in 50 min, 25–35% in 10 min. At the end of the gradient column was washed with a ramp to 80%B and re-equilibrated (A - 0.1% formic acid in water, B - acetonitrile, 0.1% formic acid, flow rate of 0.4 µL/min). An LC-MSMS consisting of a nanoAquity UPLC (Waters, MA), and a Thermo Fusion Lumos (Thermo Fisher, San Jose, CA) tribrid mass spectrometer with electrospray ionization were used for the analysis.

Data-independent analysis parameters were as follows: MS1 scan (395–1005 Da, resolution 120,000, maximum injection time 50 ms) followed with 60 MS/MS scans across 400–1000 Da range with 10 Da mass selection window each (resolution 15,000, maximum injection time 22 ms, loop time 3 sec) Fragmentation was induced by HCD activation at normalized collision energy 30%. Further data processing was accomplished using Skyline^17^ to extract fragment ion chromatograms of the MS2 scans (top 5 fragment ions for the peptides derived from proteins of interest listed in Table 2) with 10 ppm accuracy windows. Chromatograms were integrated and chromatographic peak areas were exported for further analysis. Relative quantification of Apoa1, Saa1, Saa2 and Saa3 was accomplished using the ratio of the endogenous peptide peak area to peak area of the corresponding SIL peptide (peptides specific to Saa1 and Saa2 sequence were used to distinguish these two highly homologous isoforms). Abundance of the other proteins was quantified after normalization of the representative peptides to ^15^N-APOA1 peptide VQPYLDDFQK. To quantify each protein, a response of 3 peptides specific to each protein was averaged. Peptide and protein abundance is therefore expressed in arbitrary units [a.u.]. The common denominator for plasma analysis was volume of plasma; for the FPLC fractions the denominator was equal volumes of each fraction.

#### Proteomic analysis of liver and adipose tissue

Tissue was homogenized with 8 M urea, 100mMTris, pH8.0, 0.05%SDS buffer and its protein concentration was measured by the BCA assay (Thermo Fisher Scientific). Tissue extract (40 μg) was separated on SDS-PAGE (Nupage, Thermo Fisher Scientific) and the precise localization of Saa proteins was determined by western blot (Saa1 antibody AF2948, R&D System) run in parallel on a separate gel. The section of the gel covering Saa and the adjacent area was then excised and processed for proteomics analysis by in-gel digestion. Extracts were dried and reconstituted for LC-MS/MS analysis. To increase the sensitivity of detection of Saa proteins, LC-MS/MS analysis was performed on the same instrument described earlier, using a parallel-reaction monitoring approach following the same peptides for Saa1, Saa2 and Saa3 as indicated for plasma analysis.

### Statistical analyses

Data were analyzed using GraphPad Prism 6 software and are presented as means ± standard errors unless otherwise indicated. For plasma ELISA and proteomics data, one-way ANOVA was used to compare LPS injections to the control, and unpaired t-tests were utilized for the other acute inflammation models. Two-way ANOVA was used to compare gene expression and proteomics data between tissues of mice receiving the different injections, with Sidak post-hoc testing for multiple comparisons. A p value < 0.05 was considered to be statistically significant.

## Results

### Detection of low plasma levels of Saa3 is dependent on the nature and extent of the inflammatory stimulus

**Figure 1:**
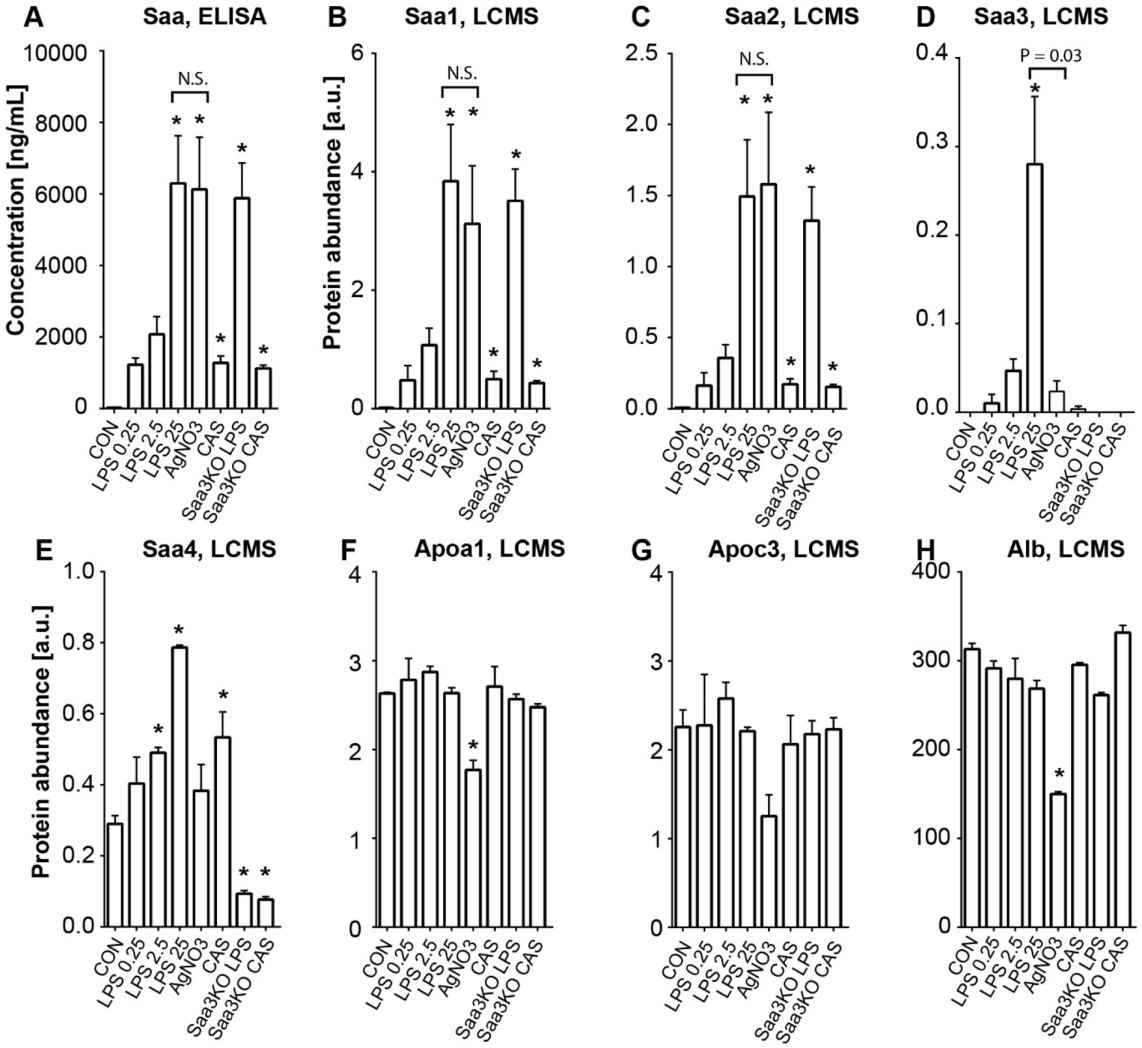
Plasma levels of Saa1, Saa2 and Saa3 in multiple models of acute inflammation. Acute inflammation was induced by LPS (0.25, 2.5, 25 µg), silver nitrate (AgNO_3_), and casein (CAS) in C57Bl6 and SAA3-KO animals. Control animals (CON) received no injections. Total plasma Saa was measured by ELISA (**A**). Saa1 (**B**), Saa2 (**C**), Saa3 (**D**) and Saa4 (**E**) were measured by LC-MS/MS. Apoa1 (**F**), Apoc3 (**G**) and albumin (**H**), measured by LC-MS/MS as controls, were only modestly affected by acute inflammation. Data are presented as mean ± SEM. n = 3 mice/group. *P < 0.05 from CON; N.S. = not significant. [a.u.] – arbitrary units.

Levels of Saa isotypes were measured by LC-MS/MS in plasma from mice inflamed by increasing concentrations of LPS, silver nitrate or casein (Table 3). All modalities of inflammation resulted in elevated levels of plasma Saa as determined by ELISA, using an antibody that detects mainly Saa1 and Saa2 (Fig. 1A). Specific measurement of Saa1, Saa2 and Saa3 by LC-MS/MS showed a dose-dependent elevation of Saa1 (Fig. 1B) and Saa2 (Fig. 1C) levels after LPS injection relative to control mice that had not received an inflammatory stimulus. Silver nitrate-induced inflammation resulted in plasma Saa1 and Saa2 levels comparable to high dose LPS (Fig. 1B,C). LPS elicited a dose-dependent increase in plasma levels of Saa3 that were about 10x lower than levels of Saa1 or Saa2 (significantly elevated only after LPS 25 µg) (Table 3). After silver nitrate injection, even lower levels of Saa3 were detected in plasma (<10% of the level observed after LPS 25 µg), despite Saa1 and Saa2 plasma levels that were comparable to those observed with LPS 25 µg (Fig. 1). Plasma Saa measured by ELISA (Fig. 1A) and Saa1 and Saa2 measured by LC-MS/MS (Fig. 1B,C) increased to a similar level in *Saa3*-deficient and control mice after injection of 25 µg of LPS. Confirming detection specificity, Saa3 was not detected by LC-MS/MS in Saa3-deficient mice (Fig. 1D). Taken together, these data suggest that Saa3 does not contribute to total plasma Saa to a major extent and that Saa3 is highly selectively induced by different inflammatory stimuli. Levels of several other apolipoproteins (Apoa1, Apoc3) and albumin also were assessed in plasma but did not show major differences from control levels in the various studied inflammatory mouse models (Fig. 1F,G), except modest reduction after injection of silver nitrate. Plasma levels of Apoa2 and Apoe were altered similarly to Apoa1. Although Saa4 is believed to be a constitutive form of Saa, it did increase slightly in response to the injection of LPS and casein (Fig. 1E), which could be reflected in a change in HDL-low density lipoprotein interactions as previously described^18^.

**Table 3:** Fold induction of Saa isotypes in plasma after various inflammatory stimuli measured by LC-MS/MS. Presented as mean ± SD fold induction relative to the no injection C57Bl/6 control mice.

	CON	LPS 0.25	LPS 2.5	LPS 25	AgNO3	CAS	Saa3KO LPS	Saa3KO CAS
Saa, ELISA	1.0	60.6	102.7	311.6*	303.3*	63.1	291.1*	55.5
	±0.29	±5.42	±14.17	±38.17	±41.72	±5.57	±28.14	±2.63
Saa1, LCMS	1.0	35.0	78.2	280.3*	227.8*	36.2	256.3*	31.4
	±0.37	±10.38	±12.18	±40.42	±41.41	±5.75	±22.67	±1.93
Saa2, LCMS	1.0	22.4	49.2	205.7*	217.6*	23.7	182.3*	21.1
	±0.12	±7.15	±7.33	±31.65	±40.24	±3.15	±18.82	±1.33
Saa3, LCMS	1.0	2.3	6.6	41.7*	4.2	1.1	N.D.	N.D.
	±0.08	±0.8	±1.23	±6.51	±0.68	±0.19		

*P < 0.05 from CON.

### Distribution of Saa3 in plasma induced by the injection of a high dose of LPS differs from that of Saa1, Saa2 and Saa4

**Figure 2:**
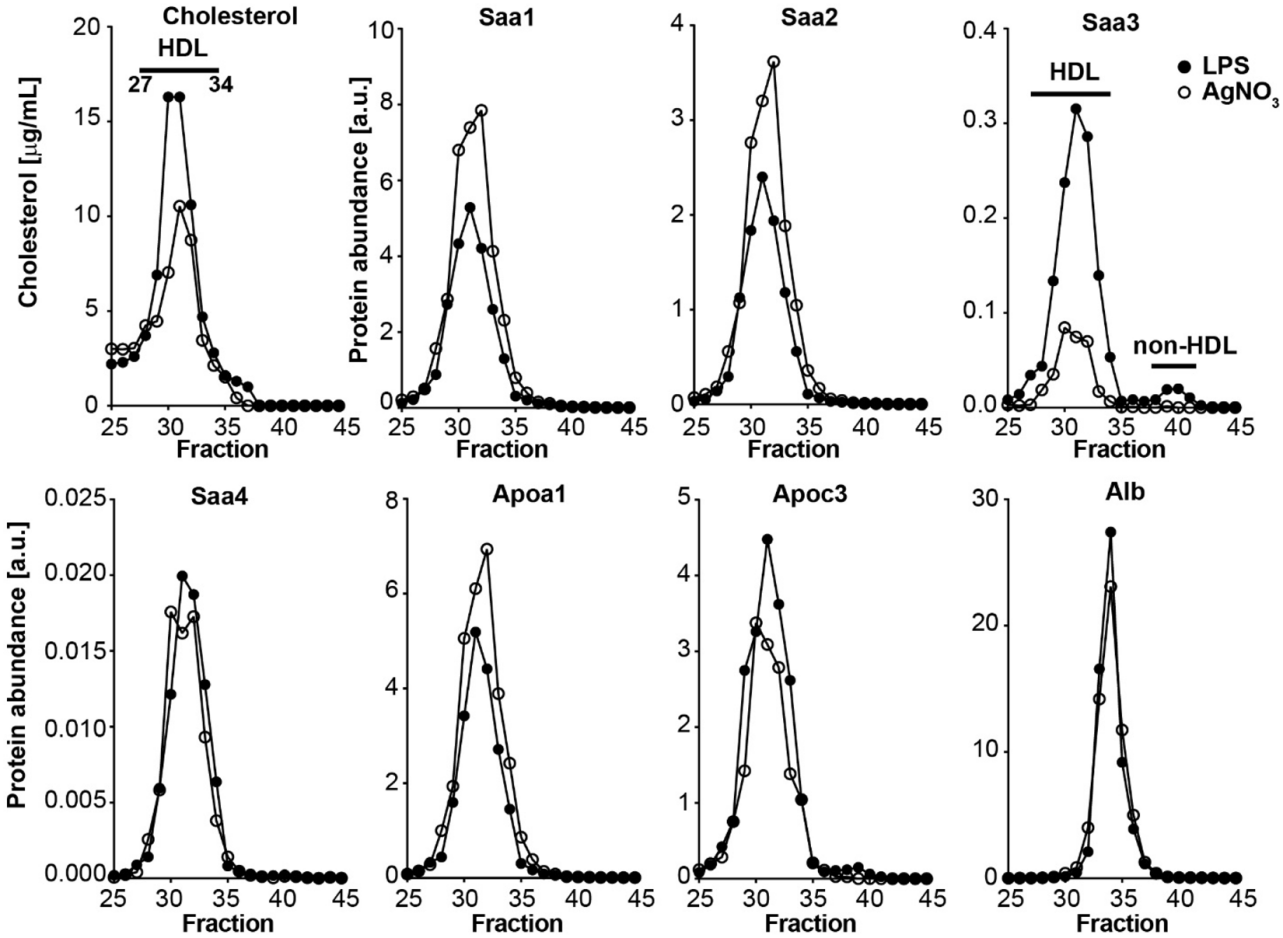
Distinct plasma distribution of Saa1, Saa2, and Saa3 measured by LC-MS/MS after acute inflammation induced by 25 µg of LPS or silver nitrate. Plasma was fractionated by FPLC and lipoprotein distribution determined based on cholesterol measurements. Relative levels of Saa1, Saa2, Saa3, Saa4, Apo1, ApoC3 and albumin were quantified by LC-MS/MS. [a.u.] – arbitrary units.

The Saa family of proteins are transported in plasma mainly associated with HDL and generally are not considered to be present in a non-lipoprotein associated form^10,11,13^. To determine whether the Saa3 that was detected in plasma after the injection of 25 µg LPS had a similar plasma distribution to the other forms of Saa, distribution of all isotypes of Saa was assessed by LC-MS/MS in FPLC fractionated plasma. While Saa1, Saa2 and Saa4 were exclusively found in fractions corresponding to HDL (Fig. 2), as has been described previously^10,11^, a small amount of Saa3 also was detected in fractions corresponding to smaller non-HDL fractions (Fig. 2) as reported by Tannock *et al*. after LPS injection^6^. After silver nitrate administration, Saa3 was undetectable in non-HDL fractions, possibly due to detection limit of the LC-MS/MS method given already low levels of Saa3 in plasma (Fig. 2).

### Saa3 present in plasma is likely derived from adipose tissue

**Figure 3:**
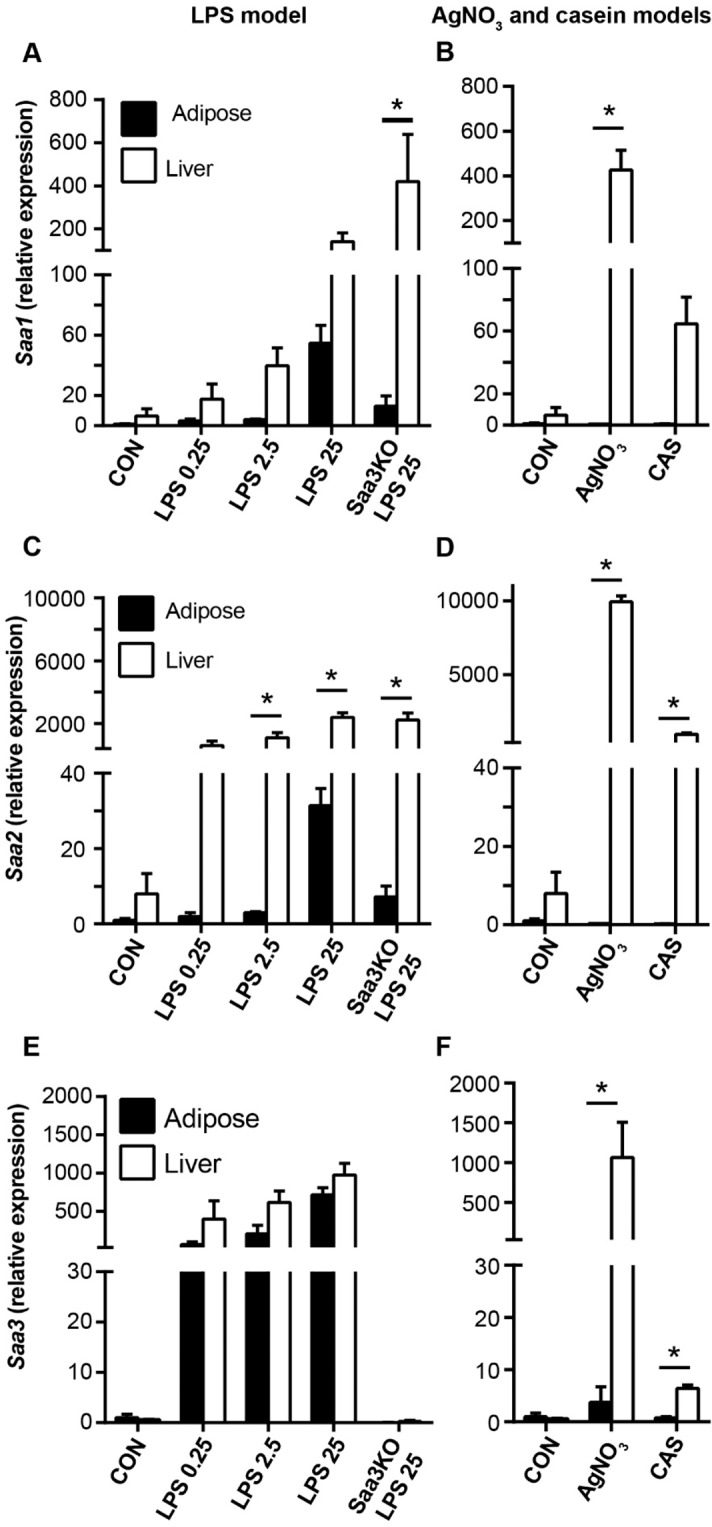
Comparison of *Saa* subtype expression between adipose and liver with different inflammatory stimuli. Gene expression of *Saa1, Saa2*, and *Saa3* was quantified from epididymal white adipose tissue (closed bars) and liver (open bars) of C57Bl/6 or *Saa3* KO mice injected with LPS (**A,C,E**), silver nitrate or casein (**B,D,F**). Data are presented as mean ± SEM. n = 3 mice/group. *P < 0.05 from EWAT.

**Figure 4:**
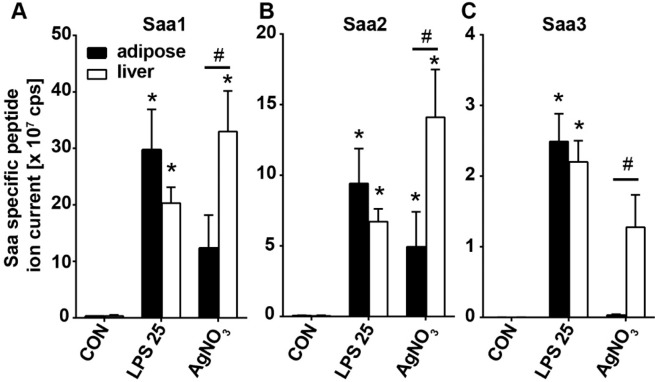
Saa3 protein is induced in adipose tissue and liver by LPS but only in liver by silver nitrate. Saa1 (**A**), Saa2 (**B**) and Saa3 (**C**) proteins were measured by mass spectrometry in the epididymal white adipose tissue (closed bars) and livers (open bars) from control mice (CON), or mice injected with 25 µg LPS (LPS 25) or silver nitrate (AgNO_3_). Data are presented as mean ± SEM (n = 3 per group). *P < 0.05 from CON. ^#^P < 0.05 between tissue types.

Gene expression analysis of the various inducible forms of Saa (*Saa1, Saa2* and *Saa3*) in liver and adipose tissue revealed striking differences that provide potential clues as to the sources of circulating Saa3. High expression levels of *Saa1* (Fig. 3A,B) and *Saa2* (Fig. 3C,D) were mainly observed in the liver in all models of acute inflammation, while in adipose tissue low expression of both these Saa isotypes was observed mainly for mice injected with 25 µg LPS (Fig. 3A,C). Expression of *Saa3* in liver was similar between mice injected with 25 µg LPS and with silver nitrate (Fig. 3E,F). In contrast, adipose tissue expression of *Saa3* was markedly higher after 0.25, 2.5, and 25 µg of LPS and was much higher than after silver nitrate, while liver expression of Saa3 was comparable between LPS and silver nitrate injected animals (Fig. 3E,F). Expression of *Saa3* in the casein-induced inflammation model (Fig. 3F) was much lower than *Saa1* and *Saa2* in the liver, and essentially undetectable in adipose tissue. The fold change of expression for each gene, tissue, and treatment is shown in Table 4. Although *Saa3* was expressed by the liver to a similar extent in mice treated with 25 µg LPS and with silver nitrate, much less Saa3 was detected in plasma from the silver nitrate injected mice. To investigate whether this may be the result of altered Saa3 protein production, we measured the amount of Saa3 protein in EWAT and liver extracts from these mice by LC-MS/MS. Similar amounts of Saa1 and Saa2 were detected in adipose tissue and in liver after both treatments (Fig. 4A,B). However, although similar amount of Saa3 protein was detectable in adipose tissue and in liver extracts after the injection of 25 µg LPS, after silver nitrate injection very little Saa3 protein was detected in adipose tissue (Fig. 4C). Collectively, these gene expression and tissue proteomic data strongly suggest that the main source of the circulating Saa3 in the LPS models is adipose tissue, with much smaller contribution from liver.

**Table 4:** Mean expression levels of *Saa1*, *Saa2*, and *Saa3* for the indicated tissues and treatments, normalized to *Gapdh* and presented as a fold change from adipocyte CON ± SEM. n = 3 mice/group.

	CON	LPS 0.25	LPS 2.5	LPS 25	Saa3 KO LPS 25	AgNO_3_	Casein
**Adipose**							
*Saa1*	1.0 ±0.4	3.1 ±1.3	4.1* ±0.2	54.6* ±12.1	12.8 ±7.0	0.5 ±0.2	0.6 ±0.4
*Saa2*	1.0 ±0.45	2.0 ±1.0	3.0* ±0.28	31.4* ±4.5	7.2 ±2.9	0.3 ±0.07	0.15 ±0.08
*Saa3*	1.0 ±0.69	65.9 ±35.1	207.8 ±113.0	717.6* ±92.0	0.05 ±0.02	3.8 ±2.9	0.8 ±0.2
**Liver**							
*Saa1*	6.4 ±4.9	18.6 ±10.1	39.8 ±11.8	141.6* ±39.4	419.2 ±220.6	426.2* ±78.6	64.6* ±17.1
*Saa2*	7.8 ±5.4	598.9 ±287.4	1109.1* ±328.5	2403.9* ±292.9	2223.8* ±453.2	9928.0* ±402.1	954.1* ±103.3
*Saa3*	0.6 ±0.05	401.8 ±235.2	616.7* ±150.0	977.4* ±151.0	0.2 ±0.2	1064.2* ±445.6	6.4* ±0.7

*P < 0.05 from CON.

## Discussion

We previously have demonstrated the presence of SAA in HDL from mice injected with silver nitrate and in HDL isolated from obese mice with moderate inflammation and humans with systemic lupus erythematosus^19^. The presence of SAA resulted in loss of the ability of HDL to blunt inflammatory stimuli^19^. However, the nature of the specific SAA isotypes was not defined in that paper.

Using a highly sensitive and specific mass spectrometric method, we also previously have shown that Saa3 could not be detected in plasma from obese mice that had chronic but modest elevations of their plasma Saa levels^5^. The inability to detect Saa3 in plasma by mass spectrometry was subsequently reported in mice in which the expression of *Saa3* was modestly increased in both adipose tissue and colonic epithelium^20^. In contrast, Tannock *et al*. reported detection of Saa3 in plasma after acute inflammation induced by a high dose of LPS^6^. In the current study we report that low levels of Saa3 appeared in plasma only after some modalities of acute inflammation, including high dose LPS injection and to a much more limited degree (~10% of the level after the high dose LPS) after sterile inflammation induced by silver nitrate injection. We were unable to detect Saa3 in plasma after casein injection. Our findings are consistent with older studies of Meek and Benditt^21^ who used a specific antibody against Saa3, as well as the more recent findings of Tannock *et al*., who used isoelectric focusing to detect Saa3^6^, both of which found Saa3 to be present in plasma after injection of a high dose of LPS.

For our study we chose to use C57Bl/6 mice fed a chow diet to minimize the chronic inflammation and modest elevations of plasma Saa levels and accumulation of macrophages in adipose tissue that occurs in obese mice^13^. Moreover, in lean chow fed mice, Saa is transported in plasma entirely in HDL^13^, as occurs in healthy human subjects^10^, but not in obese mouse models^13^ and some obese humans^14^ in which SAA can be transported in lower density lipoproteins. By using lean mice, we avoided the possible confounder of non-HDL associated subtypes. We also specifically used methods that avoided ultracentrifugation that can dissociate some loosely associated apolipoproteins from the surface of lipoproteins^22^. Because we previously have shown that Saa3 exhibits sexual dimorphic effects on body weight and inflammation^7^, and because male mice tend to become more inflamed than females, we chose to only use male mice for these studies. However, we cannot preclude that the results would be the same in female mice.

Our findings indicate that the appearance of Saa3 in plasma is dependent on both the mode of and the extent of the acute inflammatory stimulus. LPS injection, especially at a high dose, resulted in a small amount of Saa3 being detected in plasma by the highly sensitive and specific LC-MS/MS method used in this study. Lower doses of LPS led to much lower levels of Saa3, as did sterile inflammation resulting from the injection of silver nitrate, while acute inflammation induced by casein failed to result in detectable Saa3 (Fig. 1). Therefore, it is not surprising that we were unable to detect Saa3 in plasma from obese mice^5^, nor that it was not detected in mice with modest inflammation in adipose tissue and colonic epithelial cells and macrophages induced by manipulation of the intestinal microbiota^20^. In agreement with the report from Tannock *et al*. that used high-dose LPS to induce acute inflammation, we also observed the bulk of Saa3 to be present on HDL^6^, as occurs with the other isotypes of Saa. After density gradient ultracentrifugation, Tannock *et al*. were able to dissociate a relatively large amount of Saa3 from HDL, leading to the conclusion that it is more loosely associated with HDL that Saa1 or Saa2^6^. Although we did not use ultracentrifugation in our study, it is possible that the low speed centrifugation used to separate plasma might have been sufficient to displace some Saa3 from HDL, leading to our findings with FPLC. A small fraction of SAA has recently been shown to circulate bound to retinol rather than HDL in inflammatory states^23^. Since we did not measure retinol, we are unable to speculate whether the non-HDL Saa3 we observed might be bound to retinol. However, HDL-associated Saa3 appeared in larger molecular weight fractions, while free Saa3 appeared in smaller molecular fractions than retinol binding protein 4.

Adipose tissue is well known to be a source of *Saa3* expression, primarily in adipocytes. Its expression is upregulated in adipose tissue from diabetic mice^24^ and by consumption of an obesogenic diet^13^. *Saa3* expression also is increased by exposure of cultured adipocytes to high glucose concentrations^25^ and to saturated fatty acids^26^. In the current study, of all the inflammatory stimuli tested, high levels of *Saa3* expression in adipose tissue were only detected after mice were injected with LPS, in which *Saa3* expression was markedly induced in a dose-dependent manner in both liver and adipose tissue. *Saa3* expression levels in the liver were similar after the injection of 25 µg of LPS and silver nitrate. In contrast, much lower levels of *Saa3* expression were detected in adipose tissue after silver nitrate injection than after the three doses of LPS used (Fig. 3E,F). Because we could only detect Saa3 in the plasma following high-dose LPS injection, and minimally following silver nitrate injection in which high levels of *Saa3* expression were observed in the liver, it is tempting to speculate that the plasma Saa3 is derived in large part from adipose tissue. It is unclear why *Saa3*, which was expressed by the liver to an equivalent extent in mice treated with 25 µg of LPS and silver nitrate, failed to appear at similar level in plasma after silver nitrate injection. Our finding that very little Saa3 protein was detected in adipose tissue from the mice injected with silver nitrate suggests that hepatic *Saa3* mRNA stability may be low^27^, that Saa3 is poorly translated, has impaired secretion or is rapidly degraded in these mice. Alternatively, it is possible that the inflammatory mediators resulting from the local inflammation induced by silver nitrate^28,29^ do not reach the adipose tissue systemically, in contrast to a direct effect of LPS in the adipose tissue. Collectively these data suggest that adipose tissue is the major source of Saa3 in plasma after LPS injection.

Recently, an important role for macrophages in adipocyte-derived Saa3 expression has been proposed. Sanada *et al*. showed that conditioned medium from macrophages stimulated with LPS could induce Saa3 expression from adipocytes in a C/EBP-dependent manner^30^. This suggests that the recruitment of macrophages into adipose tissue by LPS may promote Saa3 expression and secretion from adipocytes by a different mechanism than from hepatocytes. It is also possible that the recruited adipose tissue macrophages are the source of adipose-derived Saa3, instead of or in addition to adipocytes. Future experiments could tease apart the adipocyte and macrophage contributions to adipose tissue-derived Saa3 in response to high dose LPS.

SAA3 is a pseudogene and is not transcribed in humans, which produce SAA1 and SAA2 in response to inflammatory stimuli in extrahepatic tissues as well as in the liver^1^. For example, SAA1 and SAA2 are expressed by tissue macrophages in several inflammatory conditions^31–33^. The question of whether adipose tissue-derived SAA contributes to the circulating pool of SAA in humans is hampered by the fact that the isotypes of SAA produced by human adipocytes and macrophages, i.e., SAA1 and SAA2, are the same as those produced by the liver^1^. Obese human subjects had high levels of *SAA1* and *SAA2* expression in adipose tissue^3,34,35^, which correlated with plasma SAA levels^34^. Both tissue mRNA expression levels and plasma SAA levels fell with weight loss^34,35^, suggesting that adipose tissue might be a source of plasma SAA in human obesity. However, it is possible that cytokines produced in adipose tissue in obesity might have activated hepatic production of SAA, which could be the source of SAA in the circulation. Development of a transgenic mouse model that specifically expresses human SAA in adipose tissue showed the presence of human SAA in plasma of these transgenic mice after high fat feeding. However, interpretation is hampered by the observation that transcript levels in adipose tissue were not increased by the diet used^36^. Our findings suggest that extrahepatic Saa produced in adipose tissue of mice can enter plasma after certain but not all acute inflammatory stimuli. This differs from the chronic inflammatory state associate with obesity, in which we previously were unable to detect Saa3 in plasma^5^.

In our study, although *Saa2* expression in response to stimuli was higher in liver than that of *Saa1*, protein levels of Saa1 and Saa2 were roughly equivalent in plasma, liver and adipose tissue. Gene expression of hepatic *Saa1* and *Saa2* in response to various inflammatory stimuli have been reported extensively^8,15^. While most studies find roughly equivalent expression levels of *Saa1* and *Saa2*, a trend toward higher *Saa2* expression levels also has been reported previously by us and others in various mouse models of inflammation^7,37^. Since we have confirmed that the primers used to distinguish *Saa1* and *Saa2* do not exhibit sequence overlap (ThermoFisher Scientific), our results suggest a mismatch between gene and protein expression levels. For gene expression levels to exceed translated protein levels is relatively common for various inflammatory cytokines such as tumor necrosis factor alpha (TNFα)^38,39^, which is transcriptionally regulated similarly to Saa. Thus, our gene expression results are in line with and expand upon what has been previously reported.

LPS-mediated inflammation is widely viewed as evoking a direct systemic effect, while the systemic inflammation elicited by silver nitrate is thought to be secondary to its local inflammatory effects^28,29^. It is thus possible that LPS and silver nitrate evoke different signaling mechanisms within different tissues. LPS induces inflammatory effects that involve the transcription factors NFkB, activating protein-1 (AP-1), and activating transcription factor 2 (ATF2)^40^, and could modulate different inflammatory effects in liver and adipose tissue. In contrast, there is evidence in mice and rabbits that silver nitrate specifically targets the liver more than other tissues, such as spleen, kidney, skeletal muscle, brain, lung, and heart^41,42^. Importantly, silver nitrate-mediated induction of hepatic Saa is accompanied by precise coordination of transcription factors, including SAA-activating factor (SAF), nuclear factor kappa-B (NFkB), and CCAAT/enhancer-binding protein (C/EBP), with different transcription factor combinations and kinetics displayed in other tissues^42^. To our knowledge transcriptional regulation of silver nitrate-induced Saa in the adipose tissue has not been reported. Indeed, previous work supports the notion that particular inflammatory stimuli such as casein and LPS can produce diverse inflammatory effects in different tissue types, resulting in different Saa isotype expression patterns^8^. Additional studies would be required to determine whether adipose and liver tissue respond differently to silver nitrate and LPS.

In summary, the appearance in plasma of different Saa isotypes after acute inflammatory stimuli depends largely on the nature and extent of the stimulus and the predominantly extrahepatic form of Saa, Saa3, only appears in plasma to any significant extent after high dose LPS, and appears to originate mainly from adipose tissue.
